# Effects of *Salvia miltiorrhiza* extract with supplemental liquefied calcium on osteoporosis in calcium-deficient ovariectomized mice

**DOI:** 10.1186/s12906-017-2047-y

**Published:** 2017-12-20

**Authors:** Bongkyun Park, Hae Seong Song, Jeong Eun Kwon, Se Min Cho, Seon-A Jang, Mi Yeon Kim, Se Chan Kang

**Affiliations:** 1 Department of Oriental Medicine and Biotechnology, College of Life Science, Kyung Hee University, Yongin-si, 17104 Republic of Korea; 2 KMF Co., Ltd., Yulam-ro 12, Dong-gu, Daegu-si, 41065 Republic of Korea

**Keywords:** *Salvia miltiorrhiza* Bunge, Liquefied calcium supplement, Ovariectomized mice, RANKL, OPG

## Abstract

**Background:**

Extracts from *Salvia miltiorrhiza* Bunge have been used in traditional Asian medicine to treat coronary heart disease, chronic renal failure, atherosclerosis, myocardial infraction, angina pectoris, myocardial ischemia, dysmenorrheal, neurasthenic insomnia, liver fibrosis and cirrhosis. The aim of the study was to investigate the anti-RANK signal effect of the combination of *S.miltiorrhiza* Bunge (SME) and liquefied calcium (LCa) supplement with ovariectomized (OVX-SML) mice, a osteoporosis animal model. Results were compared to 17β-estradiol (E_2_) treatment.

**Methods:**

A total of 70 female ICR strain mice (7 weeks) were randomly divided into 10 groups with 7 mice in each group as follows: (1) sham-operated control mice (sham) received daily oral phosphate-buffered-saline (PBS) of equal volumes through oral administration. (2) OVX mice received a daily oral administration of PBS (OVX). (3) OVX mice treated daily with 50 mg/kg b.w./ day of SME (4) with 100 mg/kg b.w./day of SME or (5) with 200 mg/kg b.w./day of SME via oral administration. (6) OVX mice treated daily with 50 mg/kg b.w./day of SML (7) with 100 mg/kg b.w./day of SML or (8) with 200 mg/kg b.w./day of SML via oral administration. (9) OVX mice treated daily with 10 ml/kg b.w./day of LCa (10) OVX mice received i.p. injections of 17β-estradiol (E_2_) (0.1 mg/kg b.w./day) three times per week for 12 weeks.

**Results:**

micro-CT analysis revealed that oral administration of SML inhibited tibial bone loss, sustained trabecular bone state, and ameliorated bone biochemical markers. In addition, SML administration compared to SEM and LCa reduced serum levels of RANKL, osteocalcin and BALP through increased serum levels of OPG and E_2_ in OVX mice. SML also had more beneficial effects on protection of estrogen-dependent bone loss through blocking expression of TRAF6 and NFTAc1 and produces cathepsin K and calcitonin receptor to develop osteoclast differentiation.

**Conclusion:**

These data suggest that *S. miltiorrhiza* Bunge combined with liquefied calcium supplement has an inhibitory activity in OVX mice. This result implies the possibility of a pharmacological intervention specifically directed toward a disease such as osteoporosis where decreased bone strength increases the risk of a broken bone.

**Electronic supplementary material:**

The online version of this article (10.1186/s12906-017-2047-y) contains supplementary material, which is available to authorized users.

## Background

Osteoporosis is often regarded as a compensatory disease for reduced bone strength and is influenced by mechanisms controlling bone remodeling [1, 2]. In women, remodeling mechanisms are significantly influenced by ovarian function and are remarkably changed by ovarian aging and reproductive damage. These signs are related to an estrogen deficiency, and not a genetic or mechanical cause [3]. In addition, it has been reported that osteoporosis can be classified as two types. Type I osteoporosis is the most common in postmenopausal women and is related to an estrogen deficiency. Type II osteoporosis is senile osteoporosis, caused by aging, and is largely due to decreased dietary calcium and vitamin D or increased parathyroid gland activity [4, 5].

Many experimental models have been indicated to induce osteoporosis. Among these models, the mice ovariectomized (OVX) model is the most well-known and popular model to investigate bone loss in proximal tibia, the distal femur, and the lumbar vertebrae [6]. In addition, FDA guidelines have been demonstrated that the characteristics of bone loss in the OVX model simulate the bone alterations following menopause or oophorectomy in human. Therefore, this model is appropriate to exploit the preventive potential of new therapeutic agents [7]. There are also some alternative animal experimental models to induce osteoporosis. In the rat model, alcohol abuse increased the pathogenesis of inhibited bone mass [8]. A high cholesterol or calcium deficient model induced osteoporosis through inhibiting osteoblast differentiation and growth [9, 10]. The diabetic related osteopenia has also been widely examined in several experimental investigation [11, 12].

Estrogen plays an essential role in bone homeostasis and skeletal growth. Estrogen deficiency induces chronic inflammatory conditions by increasing various cytokines, free radicals, and growth factors, which produce an altered bone microenvironment by increasing bone loss and osteoclast formation [4, 13–15]. Moreover, estrogen reduces intestinal calcium (Ca) absorption and Ca availability [16, 17]. Accordingly, hormone replacement therapy (HRT) has been used for postmenopausal osteoporosis. However, the long-term usage of estrogens like 17β-estradiol in postmenopausal women gives rise to various side effects, such as gastrointestinal tolerance and/or breast, ovarian, and endometrial cancers [18–20]. There has been recent interest in plant-derived compounds that have a comparable structure to estrogen and a similar ability to bind estrogen receptors [21]. The estrogen-similar effects of these compounds represent an alternative approach to replace or augment HRT for managing osteoporosis [22].

*Salvia miltiorrhiza* Bunge (SME) is a native species of a perennial plant in the genus *Salvia* from China, Japan, and Korea [23]. It has been known as a traditional herbal medicine for anti-inflammatory activity in rat carrageenan-induced paw oedema and adjuvant-induced arthritis [24], anti-oxidation activity in high fat diet induced atheroscleotic mice [25], and anti-osteoporotic efficacy on the Chinese herbal prescriptions including *Salvia miltiorrhiza* in clinical trial of menopause women [26]. The important bioactive constituents of *S. miltiorrhiza* are tanshinones, salvianolic acid B, and danshensu [27]. Tanshinone IIA and salvianolic acid B in *S. miltiorrhiza* are the major active constituents and have antioxidant effects [27]. It has also been shown that tanshinone IIA inhibits bone resorption through attenuating the differentiation and activity of osteoclasts in vitro [28] and through the association of lipid peroxidation products with DNA in liver cells [29]. Salvianolic acid B has remarkable antioxidant activity, which has been reported in cerebral and heart ischemia in vivo*;* it also decreases malondialdehyde level and inhibits brain and heart damage in vivo [30, 31].

Calcium supplements (LCa) can be classified as natural supplements derived from shells (e.g., eggshells, oyster shells, etc.) or as synthetic supplements like calcium carbonate and calcium citrate. Synthetic calcium can be produced in huge amounts at low cost but has very low solubility; therefore, the solubility of synthetic calcium supplements must be increased for more effective absorption into the body [32–34]. To this end, liquefied calcium supplements have been shown to increase bone mineral density and bone mineral content compared to insoluble calcium supplements in clinical investigations with growing rats [35, 36]. Moreover, the combination with LCa and hormone replacement therapy (HRT) has been demonstrated that the treatment of postmenopausal women with low bone mass or bone fracture history [22].

Based on these studies, the combinational effect of SME and LCA on osteoporosis has not yet been demonstrated. The present study suggests that *S. miltiorrhiza* Bunge (SME) extract combined with liquefied calcium supplement (LCa) may have anti-osteoporotic activity in improving bone mineral density. To evaluate the therapeutic potential of a combination of SME and LCa to treat postmenopausal osteoporosis, we investigated the inhibitory effect of a combination of SME and LCa on ovariectomized mice used as a postmenopausal model.

## Methods

### Preparation of sample extracts

Dried *S. miltiorrhiza* Bunge was provided by Kyemyeong Foodex Co. (Daegu, Korea). The plant was identified and authenticated on the basis of its macroscopic and microscopic characteristics, and validated using HPLC as outlined in the Supplemental Experimental Procedures (Additional file 1: Fig. S1). Chemical analysis with thin layer chromatography (TLC) was employed in order to establish the constant composition of *S. miltiorrhiza* Bunge in accordance with the Korean Pharmacopoeia (KP), a statute for pharmaceuticals established by the Korean government for improvement of public health. *S. miltiorrhiza* Bunge (1000 g) (SME) was extracted with water at room temperature. Liquefied calcium supplements (LCa) (60 mg/ml, over 90% ionizing ratio) were prepared from calcium materials (seaweed powder, egg shells, and oyster shells) by soaking in vinegar [37] and were provided by Kyemyeong Foodex Co. (Daegu, Korea) [32]. The combination (SML) of SME and LCa was composed in demineralized water (SME 50, 100, 200 mg/ body weight (kg)/day; LCa 10 ml/body weight (kg)/ day, concentration 60 mg/kg).

### Animal care

Female 6-week-old outbred ICR mice weighing 20–23 g were purchased from Korea Laboratory Animal Co. (Daejeon, Korea) and were housed for 7 days prior to the experiment. They were housed in solid-bottomed plastic cages designed to allow easy access to standard laboratory food and water. Mice were kept in sanitary ventilated animal rooms with a controlled temperature (25 ± 1 °C) and regular light cycle (12 h light: 12 h dark). The body weight of mice was monitored every 3 days for 12 weeks, and the mice were sacrificed by CO_2_ to measure weight of uterus, thymus and spleen. Animal experiments were conducted in accordance with the current ethical regulations for animal care and use at Kyung Hee University (KHUASP(SE)-16–003).

### Postmenopausal osteoporosis induced by ovariectomy (OVX)

Mice were surgically ovariectomized under anesthesia with tiletamine/zolazapam (Virbac Korea, Seoul, Korea) and xylazine HCl (Bayer Korea, Kyungkido, Korea) using a ventral approach. At 7 weeks of age, mice were randomly divided into 10 groups of 7 mice as follows: (1) sham-operated control mice (sham) received daily oral phosphate-buffered saline (PBS) of equal volume. (2) OVX mice received a daily oral administration of PBS (OVX). (3) OVX mice treated daily with 50 mg/kg b.w. of SME, (4) with 100 mg/kg b.w. of SME, or (5) with 200 mg/kg b.w. of SME via oral administration. (6) OVX mice treated daily with 50 mg/kg b.w. of SML, (7) with 100 mg/kg b.w. of SML, or (8) with 200 mg/kg b.w. of SML via oral administration. (9) OVX mice treated daily with 10 ml/kg b.w. of LCa. (10) OVX mice received i.p. injections of 17β-estradiol (E_2_) (0.1 mg/kg b.w.) three times per week. All mice received their respective treatment for 12 weeks. At the end of the treatment period, the mice were subjected to micro-CT analysis and were sacrificed at the designated time point to provide adequate serum for assays.

### Micro-CT bone analysis

To evaluate structural loss in bone architecture, the proximal and distal parts of the right tibias were scanned by in vivo micro-computed tomography (Micro-CT, Skyscan1076, Skyscan, Antwerp, Belgium). The scan conditions were set at an aluminum filter of 0.5 mm, X-ray voltage of 50 Kv, X-ray current of 200 mA, and exposure time of 360 ms. During each scan, mice were maintained under anesthesia via inhalation with isoflurane (Hana Pharm, Seoul, Korea). Mice were placed in a chamber filled with 5% isoflurane in oxygen for 5 min, after which the isoflurane was adjusted to 1.5% to maintain anesthesia [38]. The data were then digitized using a frame grabber, and the resulting images were transmitted to a computer for analysis using Comprehensive TeX Archive Network (CTAN) topographic reconstruction software. Total volume indicated the inner area of cortical bone. Trabecular bone volume indicated the total trabecular bone within the total volume. Bone volume percentage was calculated by dividing the trabecular bone volume by the total volume. The measured cortical bone parameters were bone volume fraction (BV), mean polar moment of inertia (MMI), cross section thickness (Cs.Th), and bone mineral density (BMD). The measured trabecular bone parameters were bone volume fraction (BV/TV), trabecular thickness (Tb.Th), trabecular separation (Tb.Sp), trabecular number (Tb.N), trabecular bone pattern factor (Tb.Pf), specific bone surface (BS/BV), structure model index (SMI), and bone mineral density (BMD).

### Serum analysis

At sacrifice, samples of whole blood were collected by cardiac puncture, and blood was allowed to clot for 30 min. Serum was then separated via centrifugation at 1500 g for 10 min. Markers of bone turnover present in the serum were measured using enzyme linked immunosorbent assay (ELISA) kits for RANKL and OPG (R&D Systems, Minneapolis, MN, USA) and for E_2_, osteocalcin, and BALP (Biomedical Technologies Inc., Stoughton, MA, USA). All ELISA procedures were performed according to the manufacturers’ protocols.

### Isolation of total RNA and quantitative real-time PCR

Following the manufacturer’s protocol, total RNA was extracted from bone marrow isolated from OVX mice using Trizol Reagent. Isolated RNA (1 mg/ml) was reverse transcribed using a SuperScript II kit for cDNA synthesis. The cDNA was subjected to quantitative real-time (qRT)-PCR using thermocyclers from Applied Biosystems (Franklin Lakes, NJ, USA). For each RNA sample, the expression of β-actin was quantified by real-time RT-PCR, and a Ct method was used to estimate the differential gene expression between samples. Oligonucleotide sequences of primers used for RT-PCR using SYBR Green technology were as follows: Cathepsin K forward (5’-CACCCAGTGGGAGCTATGGAA-3′) and Cathepsin K reverse (5’-GCC TCCAGGTTATGGGCAGA-3′); Calcitonin receptor (CalcR) forward (5’-AGGCAGACCCAAATG CTGTAATG-3′) and Calcitonin receptor reverse (5’-TTGGTGATAGGTTCTTGGTGACCTC-3′); TRAF6 forward (5’-TTAAATGTCGGCATTCTCAGGGTA-3′) and TRAF6 reverse (5’-TTGTGACCGAGACTC TCC CAAG-3′); NFATc1 forward (5’-GCT TCACCCATTTGCTCCAG- 3′) and NFATc1 reverse (5’-ATGGTGTGGAAATACGGTTGGTC-3′); β-actin forward (5’-TCACCCACACTGTGCCCATCTACGA-3′) and β-actin reverse (5’-GGATGCCACAGGATTCCATACCCA-3′).

### Statistical analysis

Each result is reported as mean ± standard error of the mean (SEM). One-way analysis of variance (ANOVA) was used to determine the significance between groups, after which a modified t-test and two-way ANOVA and the Newman-Keuls test were used to analyze differences among OVX groups. Significant differences were stated for *p* values. (*p* < 0.05).

## Results

### Effects of SML on body weight and uterus weight in OVX mice

**Fig. 1:**
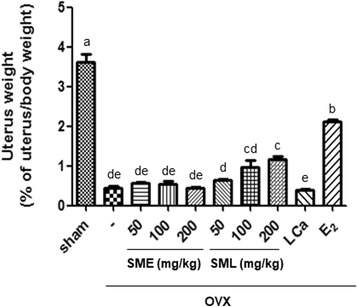
Effects of SML on body weight and uterus weight in OVX mice. The uterus weights were collected at the conclusion of the study. Values are given as mean ± S.E.M. of three independent experiments (*n* = 7) for all groups. Means unlike letters in a column with differ significantly (*p* < 0.05)

We investigated the effects of *S. miltiorrhiza* combined with liquefied calcium supplement (SML) in vivo using OVX mice. Rates of weight change were monitored every week. As shown in Table 1, body weight was significantly increased in the OVX group. However, oral administration of 200 mg/kg SML and treatment with E_2_ attenuated the increased body weight. In addition, uterine weight and uterine/body weight ratio in the OVX group were remarkably decreased compared to the sham group. Oral administration of SML at 100 or 200 mg/kg and treatment of E_2_ significantly increased the uterine/body weight ratio. Therefore, SML administration affected the body and uterine weights in OVX mice (Fig. 1). We next examined the effects of SML on the immune system. The weights of the thymus and spleen were significantly influenced by removal of the ovaries. The thymus weight in the OVX group was considerably increased compared to that in the sham group. However, it was suppressed by oral administration of SML at 100 or 200 mg/kg and by E_2_ treatment. In contrast, the spleen weight in the OVX group was decreased but was restored by oral administration of SML at 100 or 200 mg/kg and by E_2_ treatment in OVX mice (Table 2).

**Table 1:** Effects of SML on body weight in OVX mice

Period Groups		Body weight (g)			Body Weight Gains During Treatment (g)
		At OVX^α^	At Initial Treatment^α^	At Sacrifice^α^	
Sham		23.14 ± 1.23^b^	24.57 ± 0.96^c^	26.71 ± 2.51^d^	2.14 ± 1.1^cd^
OVX	–	24.29 ± 2.1 ^a^	29.67 ± 1.15 ^a^	36.59 ± 1.98^a^	6.92 ± 1.8 ^ab^
	SME 50 mg/kg	24.37 ± 1.53 ^a^	28.93 ± 0.82^ab^	35.71 ± 1.15^ab^	6.78 ± 1.17^ab^
	SME 100 mg/kg	24.1 ± 1.62 ^a^	28.71 ± 0.96^ab^	35.95 ± 1.98^ab^	7.24 ± 0.82^a^
	SME 200 mg/kg	23.94 ± 0.95^ab^	28.5 ± 0.84^b^	36.1 ± 2.23^a^	7.6 ± 1.94^a^
	SML 50 mg/kg	24.22 ± 1.15 ^a^	29.14 ± 1.11^a^	35.74 ± 1.92^ab^	6.6 ± 1.3^b^
	SML 100 mg/kg	24.41 ± 1.42 ^a^	28.59 ± 1.26^ab^	36.25 ± 2.68^a^	7.7 ± 2.21^a^
	SML 200 mg/kg	24.37 ± 1.27 ^a^	28.63 ± 1.29^ab^	32.59 ± 2.11^b^	3.96 ± 2.05^c^
	LCa 10 ml/kg	24.51 ± 0.85^a^	27.92 ± 2.21^bc^	34.93 ± 1.29^ab^	7.01 ± 1.82^a^
	E2 0.1 mg/kg	24.62 ± 1.09^a^	28.15 ± 1.42^b^	30.21 ± 1.38^c^	2.06 ± 2.13^d^

Values are given as mean ± S.E.M. *n* = 7 for all groups. ^α^All animals were overnight fasted; Means with different superscripts (a, b, c, d) are significantly different at *p* < 0.05 in pairwise comparisons

**Table 2:** Effect of SML on thymus and splenic weight in OVX mice

Parameter (mg)		OVX								
			SME (mg/kg/day)			SML (mg/kg/day)				
	sham	–	50	100	200	50	100	200	LCa (60 mg/kg/day)	E2 (0.1 mg/kg/every other day)
Thymus	49.4 ± 8.4^ab^	56.7 ± 10.5^a^	53.0 ± 9.4 ^ab^	55.9 ± 11.5^a^	54.7 ± 10.5^a^	55.7 ± 11.5^a^	52.1 ± 5.9 ^ab^	50.4 ± 8.9 ^ab^	54.3 ± 12.1^a^	47.5 ± 7.5^b^
Spleen	151.9 ± 11.5^a^	97.3 ± 13.5 ^c^	96.5 ± 13.7 ^c^	98.5 ± 13.4 ^c^	99.5 ± 19.3^c^	99.5 ± 14.7 ^c^	134.9 ± 11.5^b^	136.2 ± 15.7^b^	99.3 ± 10.5^c^	137.5 ± 12.8^b^

Values are given as means ± S.E.M. *n* = 7 for all groups. Data are the means ± S.E.M from three independent experiments. Means with different superscripts (a, b, c) are significantly different at *p* < 0.05 in pairwise comparisons.

### Effects of SML on the structural characteristics of cortical bone

**Fig. 2:**
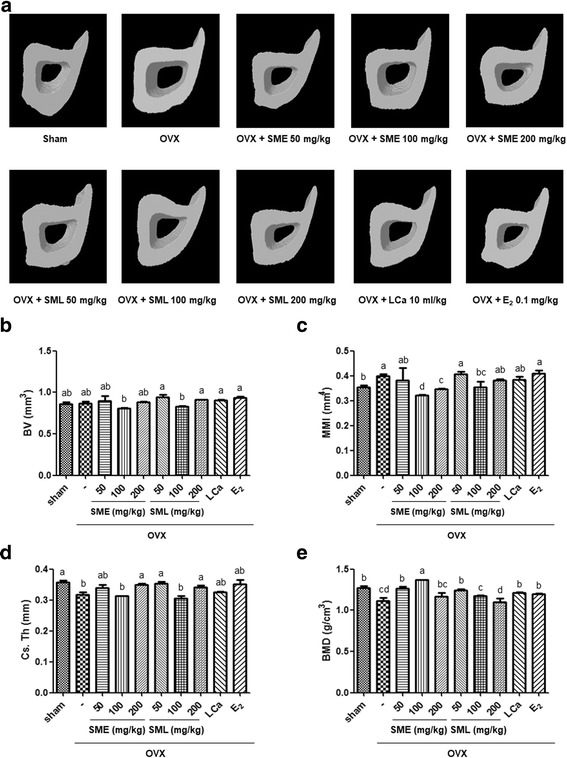
Effects of SML on the structural characteristics of cortical bone. **a** Three-dimensional micro-CT images of cortical bone after 12 weeks in OVX mice. The cortical bone parameters were measured by a three-dimensional micro-CT analysis program. The measured cortical bone parameters were **b** bone volume density (BV, mm^3^), **c** mean polar moment of inertia (MMI, mm^4^), **d** cross-section thickness (Cs.Th, mm), and **e** bone mineral density (BMD, g/cm^3^). Values are given as the mean ± S.E.M. of three independent experiments (n = 7) for all groups. Means unlike letters in a column with differ significantly (*p* < 0.05)

To determine the effects of SML on OVX-induced cortical bone structural properties, the tibia of each mouse was measured for structural parameters. Bone volume density (BV, mm^3^), mean polar moment of inertia (MMI, mm^4^), cross-section thickness (Cs.Th, mm), and bone mineral density (BMD, g/cm^3^) were scanned and calculated using micro-CT images (Fig. 2a). The measurements of Cs.Th, BV, MMI, and BMD did not change between the sham group and the OVX group. Moreover, the structural parameters were not significantly changed by oral administration of SML as well as each extract (Fig. 2 b, c, d and e).

### Effects of SML on the mechanical characteristics of trabecular bone

**Fig. 3:**
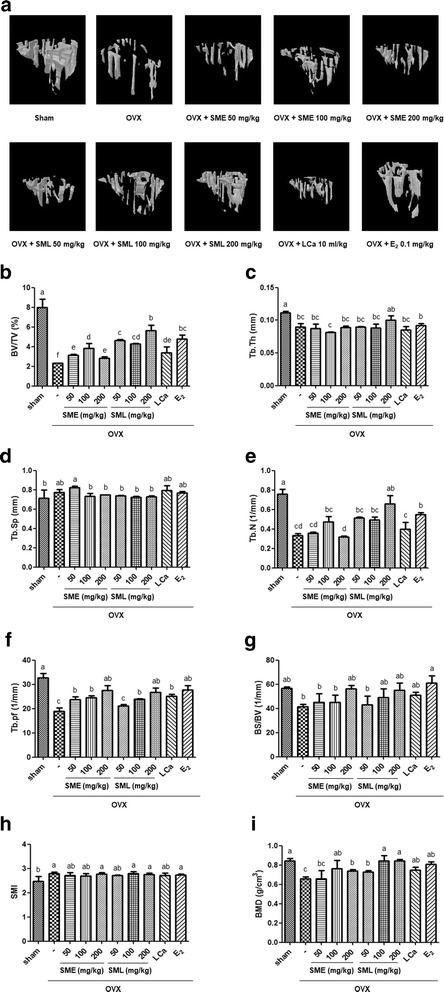
Effects of SML on the mechanical characteristics of trabecular bone. **a** Three-dimensional micro-CT images of trabecular bone after 12 weeks in OVX mice. The trabecular bone parameters were monitored by a three-dimensional micro-CT analysis program. The measured trabecular bone parameters were **b** bone volume fraction (BV/TV), **c** trabecular thickness (Tb.Th), **d** trabecular separation (Tb.Sp), **e** trabecular number (Tb.N), **f** trabecular bone pattern factor (Tb.Pf), **g** specific bone surface (BS/BV), **h** structure model index (SMI), and **i** bone mineral density (BMD). Values are given as mean ± S.E.M. of three independent experiments (n = 7) for all groups. Means unlike letters in a column with differ significantly (*p* < 0.05)

Alteration of trabecular structure is mainly mediated in postmenopausal osteoporosis, and it relates to bone strength [39]. To investigate the effects of SML on trabecular bone structure, we estimated trabecular architectural parameters from 3D micro-CT images (Fig. 3a). In the OVX group, bone volume density (BV/TV, %), trabecular thickness (Tb.Th, mm), trabecular number (Tb.N, 1/mm), trabecular bone pattern factor (Tb.Pf, mm^−1^), bone surface/volume (BS/BV, 1/mm), and bone mineral density (BMD, g/cm3) were significantly decreased (Fig. 3b–i). However, trabecular separation (Tb.Sp) and structure model index (SMI) were not altered compared to the other groups (Fig. 3d and h). Compared to the OVX group, BV/TV was significantly increased by SML administration in a dose-dependent manner (Fig. 3b). Tb.Th was only enhanced by oral administration of SML at 200 mg/kg (Fig. 3c). Tb.N was remarkably increased by SML in a dose-dependent manner, and in the E_2_-treated group (Fig. 3e). Tb.Pf and BS/BV were significantly increased by oral administration in the SME group with 200 mg/kg, in the SML group with 200 mg/kg, in the LCa group, and in the E_2_-treated group (Fig. 3f and g). In addition, BMD was reasonably restored in most groups (except on oral administration of SME at 100 or 200 mg/kg or of LCa) compared to the OVX group (Fig. 3i). However, Tb.Sp and SMI were significantly altered compared with the OVX group (Fig. 3d and h). Moreover, a high dose (200 mg/kg) of SML showed the most significant recovery effect on OVX-induced architectural deterioration of the trabecular bone.

### Effects of SML on serum biochemical markers in OVX mice

**Fig. 4:**
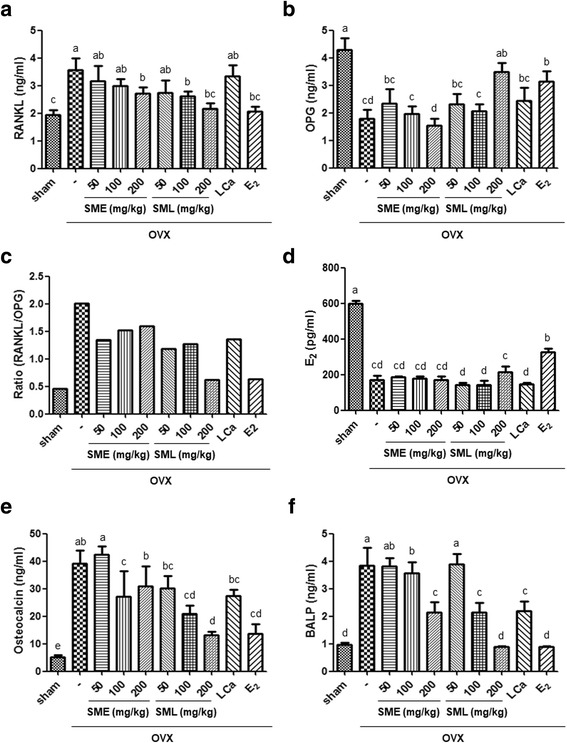
Effects of SML on serum biochemical markers in OVX mice. **a** RANKL and **b** OPG were determined by murine RANKL and OPG ELISA assays using serum, and **c** the RANKL/OPG ratio was calculated. **d** E_2_, **e** osteocalcin, and **f** BALP were determined by murine ELISA assays. Data are the mean ± S.E.M. of three independent experiments (n = 7) for all groups. Means unlike letters in a column with differ significantly (*p* < 0.05)

To demonstrate the development of osteoporosis, the following markers were suggested: RANKL, OPG, osteocalcin, BALP, and 17β-estradiol (E_2_). In addition, we investigated the inhibitory effects of SML on bone resorption-related serum markers in OVX mice. The balance between RANKL and OPG during osteoporosis is important for osteoclast regulation. This present study examined serum levels of RANKL and OPG using ELISA. In the OVX group, the serum level of RANKL was significantly increased (Fig. 4a), but OPG was suppressed compared to the sham group (Fig. 4b). Oral administration of SML at 100 and 200 mg/kg markedly decreased RANKL level compared to the OVX group (Fig. 4a). In contrast, OPG serum level significantly increased in the SML group at 200 mg/kg (Fig. 4b). The RANKL/OPG ratio was highly reduced compared to the OVX group (Fig. 4c). Moreover, E_2_ treatment significantly affected the increased serum level of OPG via inhibition of RANKL, while LCa administration did not have any effect on RANKL or OPG level compared to the OVX group. We next investigated whether SME and SML play a similar role to estrogen in OVX mice. As shown in Fig. 4d, the 17β-estradiol serum level was increased by SML at 200 mg/kg.

### Effects of SML on serum levels of osteocalcin and BALP in OVX mice

To evaluate the effects of SML on bone turnover in OVX mice, we measured the serum levels of osteocalcin and BALP by ELISA. OVX significantly increased osteocalcin level compared to the sham group. Oral administration of SME at 100 or 200 mg/kg markedly inhibited osteocalcin level (Fig. 4e). Bone alkaline phosphatase (BALP) is synthesized by osteoblasts and is presumed to be involved in the calcification of bone matrix [40]. We examined whether SML regulate BALP level using ELISA. As shown in Fig. 4f, oral administration of SME at 200 mg/kg, of SML at 100 or 200 mg/kg, of LCa, and of E_2_ suppressed the production of BALP, suggesting an effect of SML on inhibition of bone turnover and calcification of bone matrix.

### Effects of SML on the expression of cathepsin K and calcitonin receptor (CalcR) in OVX mice

**Fig. 5:**
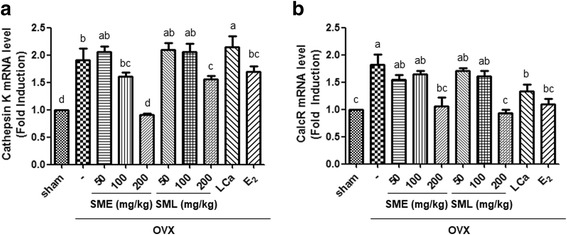
Effects of SML on the expression of cathepsin K and calcitonin receptor (CalcR) in OVX mice. RNA was extracted from the bone marrow cells of OVX mice, and **a** the mRNA levels of cathepsin K and **b** calcitonin receptor were assessed by real-time PCR assay. β-actin was considered an internal control. Data are the mean ± S.E.M. of three independent experiments (*n* = 7) for all groups. Means unlike letters in a column with differ significantly (*p* < 0.05)

Cathepsin K has been reported to be a cysteine protease involved in the bone remodeling and resorption related to osteoporosis [41]. In addition, calcitonin receptor is expressed during osteoclast differentiation to osteoblast [42]. To determine whether SML regulate bone resorption activity via the attenuation of osteoclast differentiation, we investigated the expression of the osteoclast-specific markers cathepsin K and CalcR in bone marrow cells derived from OVX mice. The mRNA expression of cathepsin K and CalcR was significantly increased in the OVX group compared to the sham group. However, oral administration of the highest doses of SME and SML and E_2_ treatment significantly inhibited the OVX-induced expression of cathepsin K and CalcR (Fig. 5a and b).

### Effects of SML on the expression of TRAF6 and NFATc1 in OVX mice

**Fig. 6:**
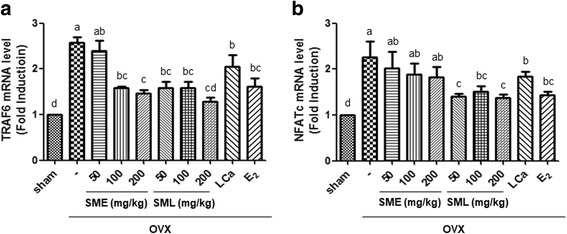
Effects of SML on the expression of TRAF6 and NFATc1 in OVX mice. RNA was extracted from the bone marrow cells of OVX mice, and **a** the mRNA levels of TRAF6 and **b** NFATc1 were assessed by real-time PCR assay. β-actin was considered an internal control. Data are the mean ± S.E.M. of three independent experiments (*n* = 7) for all groups. Means unlike letters in a column with differ significantly (*p* < 0.05)

To examine the molecular mechanism underlying the inhibitory effects of SML on osteoclast differentiation, we tested osteoclast differentiation-related signaling molecules. In the bone marrow cells of the OVX group, the mRNA expression of TRAF6 and NFATc was robust. Oral administration of SME at 100 or 200 mg/kg and SML in a dose-dependent manner significantly inhibited OVX-induced TRAF6 mRNA expression (Fig. 6a). In addition, OVX-induced NFATc expression was markedly attenuated by SML in a dose-dependent manner (Fig. 6b).

## Discussion

*Salvia miltiorrhiza* Bunge has been shown to have anticancer, antioxidant, and anti-inflammatory activities [43–45]. In addition, the ethanol extract of *Salvia miltiorrhiza* Bunge has played a role as an anti-osteoporotic agent [46]. In this study, we demonstrated that administration of SML, a combination of aqueous extract of *S. miltiorrhiza* Bunge (SME) and liquefied calcium supplement (LCa), inhibited OVX-induced osteoporosis. SML administration showed an increase of BV/TV consistent with an improvement in the BMD of trabecular bone, suggesting that it can prevent the loss of bone mass induced by OVX. In addition, Tb.N related to trabecular connectivity was restored in a dose-dependent manner by SML. Moreover, the BS/BV and Tb.Pf decreased by OVX were increased by SML administration. However, SML did not affect Tb.Sp and SMI in trabecular bone. In the cortical compartment analysis, supplementation with SML did not affect BV, MMI, Cs.Th, or BMD. We also examined the combined effects of SME and LCa on OVX-induced osteoporosis. Although administration of SME at 200 mg/kg without LCa had a mild anti-osteoporotic effect, it did not compare to that of SML. The comparison between LCa sole administration and LCa combined with SME showed that SME may have a synergistic effect on LCa supplementation, enhancing bone metabolism.

It has been reported that the main constituents of *S. miltiorrhiza* include tanshinones, salvianolic acid B, and danshensu [27]. In 2004, Kim et al. demonstrated that tanshinone IIA (known already as an antioxidant) inhibited bone resorption by attenuating the differentiation and activity of osteoclasts in vitro. In addition, it has been shown that tanshinone IIA, as a phytoestrogen, is capable of activating the estrogen receptor signaling pathway. This leads to increased endothelial nitric oxide synthase gene expression, increased nitric oxide production, and Ca^2+^ mobilization to treat cardiovascular disease [47]. Based on previous studies, phytoestrogens (such as tanshinone IIA) and isoflavones may directly interact with estrogen receptors in bone and affect bone metabolism [48, 49]. Therefore, it is possible that estrogen-like chemicals (such as the tanshinone IIA contained in *S. miltiorrhiza*) can improve intestinal Ca absorption through an estrogen-like mechanism. However, the mechanisms by which *S. miltiorrhiza* increases intestinal Ca absorption have not been elucidated, and further investigation is required.

Estrogen exerts its regulatory effects on gene expression in target tissues by different mechanisms. The uterus is one of the most estrogen-responsive reproductive tissues, and it predominantly expresses ERα [50–52]. In this study, OVX mice showed initial atrophy of the uterus, while the treatment with estradiol recovered the weight loss of the uterus. Moreover, it was found that SML administration at 100 or 200 mg/kg significantly increased the weight of the uterus. Even though *S. miltiorrhiza* Bunge combined with liquefied calcium supplement was announced to block bone loss in ovariectomized mice caused by estrogenic effect, there are some argument about whether the estrogenic effect on the gain of uterus weight might lead to develop a risk of endometrial cancer [46]. Our research could demonstrate that the administration of SML at the dose of 200 mg/kg·body weight is non-toxic effect in the OVX mice. If it alters our animal experimental dose to human dose, this supplemental dose can be converted to 973.2 mg/day for an adult having 60 kg·body weight. Accordingly, it could comfortably be taken in extract form by human. Whereas, further research is certainly required for human to have benefit effect of *S. miltiorrhiza* Bunge on osteoporosis.

It has been announced that hematopoiesis is one of the main effects of deficient estrogen on the immune system [53]. Ovariectomy is reported to increase the thymic weight, contributing to increased thymic T cell output and exacerbating bone loss, whereas splenic weight is inhibited. Therefore, E_2_ treatment is known to regulate the weight of the thymus and the spleen [54–56]. Our data supported these findings by showing an increase in the weight of the thymus in OVX mice, which was decreased by E_2_. In addition, SML administration at 100 or 200 mg/kg inhibited the thymus weight by about 8.1% (or 11.1%) in OVX mice. However, the decreased weight of the spleen in OVX mice was restored to about 27.8% (or 28.5%) on administration of SML at 100 or 200 mg/kg compared to the OVX group. The present data indicated that the combination of SME and LCa could regulate the weight of thymus and spleen to inhibit OVX-increased immune cells to develop osteoporosis.

RANKL/OPG represent a pair of cross-talk regulators between osteoblasts and osteoclasts that together regulate osteoclastogenesis [57]. Overexpression of the OPG gene in mice led to high bone mass and significant reductions in osteoclast number and activity. Additionally, this has been observed in the non-lamellated bone of OPG knockout mice [58]. RANKL is a type II transmembrane protein that exists in both membrane-bound and soluble forms [59]. It has been reported that soluble and membrane-bound RANKL are biologically active in promoting osteoclast formation [60, 61]. The pharmacological RANKL inhibition in OVX mice models, Therefore, using OPG-Fc administered immediately after the OVX procedure, was associated with increased bone volume and density and increased the biomechanical strength of the vertebrae and the femur neck sites that are prone to fragility fractures in women with postmenopausal osteoporosis [62]. In the present study, we demonstrated that SML significantly increased the levels of biochemical markers related to bone metabolism by increasing the production of OPG at the highest dose and attenuating the expression of RANKL in OVX mice. Moreover, osteocalcin as a bone turnover marker in serum is known to be up-regulated in osteoporosis and inhibited by estrogen [63]. Our study showed that OVX-induced osteocalcin level in serum was significantly reduced, but the decreased level of E_2_ caused by OVX was markedly increased (about 17%) on SML administration at 200 mg/kg compared to the OVX group. As mentioned above, increased RANKL in OVX mice exerted osteoclast differentiation. To determine whether the combination of SME and LCa inhibit osteoclast differentiation, we investigated expression of the osteoclast differentiation markers cathepsin K and calcitonin receptor (CalcR) in bone marrow cells derived from OVX mice. In the present study, we showed that OVX-induced cathepsin K and calcitonin receptor were significantly inhibited on administration of SML at the highest dose.

Bone formation markers are direct or indirect products of osteoblasts in each phase of development. They reflect different aspects of osteoblast function and bone formation and are mostly measured in the blood. Alkaline phosphatase (ALP), one of the bone formation markers, is an enzyme that plays an important role in osteoid formation and calcification. In particular, bone alkaline phosphatase (BALP) is reported to be a widely available marker of metabolic bone diseases and is increased in the serum of postmenopausal women [64]. We demonstrated that oral administration of SME at 200 mg/kg, SML at 100 or 200 mg/kg, LCa sole administration, and E_2_ treatment suppressed the production of BALP, suggesting that SML influenced on inhibition of bone turnover and calcification of bone matrix.

The ligation of RANK with RANKL results in trimerization of RANK and recruitment of adapter molecules such as the TNF receptor associated factor (TRAF) family of proteins, among which TRAF6 has been shown to be a major adapter molecule [65]. In addition, the TRAF6-mediated signaling complex has been reported to be implicated in the RANKL-stimulated activation of NFATc1, a major regulator of osteoclast-specific genes such as cathepsin K, calcitonin receptor, tartrate-resistant acid phosphatase (TRAP), osteoclast-associated receptor (OSCAR), and β3 integrin; it is often accompanied by other transcription factors such as AP-1, PU.1, microphthalmia-associated transcription factor (MITF), and CREB during osteoclastogenesis [66, 67]. In this study, the increased mRNA expression of TRAF6 and NFATc1 in OVX mice was significantly attenuated by administration of SML at the highest doses. Moreover, SML administration inhibited the mRNA expression of TRAF6 and NFATc1 in the RANK signaling pathway and inhibited the expression of cathepsin K and calcitonin receptor, leading to the inhibition of osteoclast differentiation.

Estrogen deficiency induces TNF-α secretion by T cells through a complex pathway involving the thymus and bone marrow. TNF-α upregulates osteoclast formation by increasing stromal cell production of RANKL and M-CSF [68, 69]. This situation induces an imbalance between RANKL and OPG. Additionally, it is critical in the occurrence of postmenopausal bone loss [70]. Many studies have also reported that reactive oxygen species (ROS) may play a role in postmenopausal bone loss by creating a more oxidized bone microenvironment [71]. *S. miltiorrhiza* contains many constituents such as tanshinones, salvianolic acid B, and danshensu, all of which have antioxidant activity to reduce ROS. Tanshinone IIA plays the role of a phytoestrogen in upregulating ER signaling pathways. In the present study, we demonstrated that an aqueous extract of *S. miltiorrhiza* (SME) or liquefied calcium supplement (LCa) had a partial anti-osteoporosis effect in OVX mice. However, a combination (SML) of SME and LCa had a synergistic activity that may be useful to treat osteoporosis.

## Conclusion

**Fig. 7:**
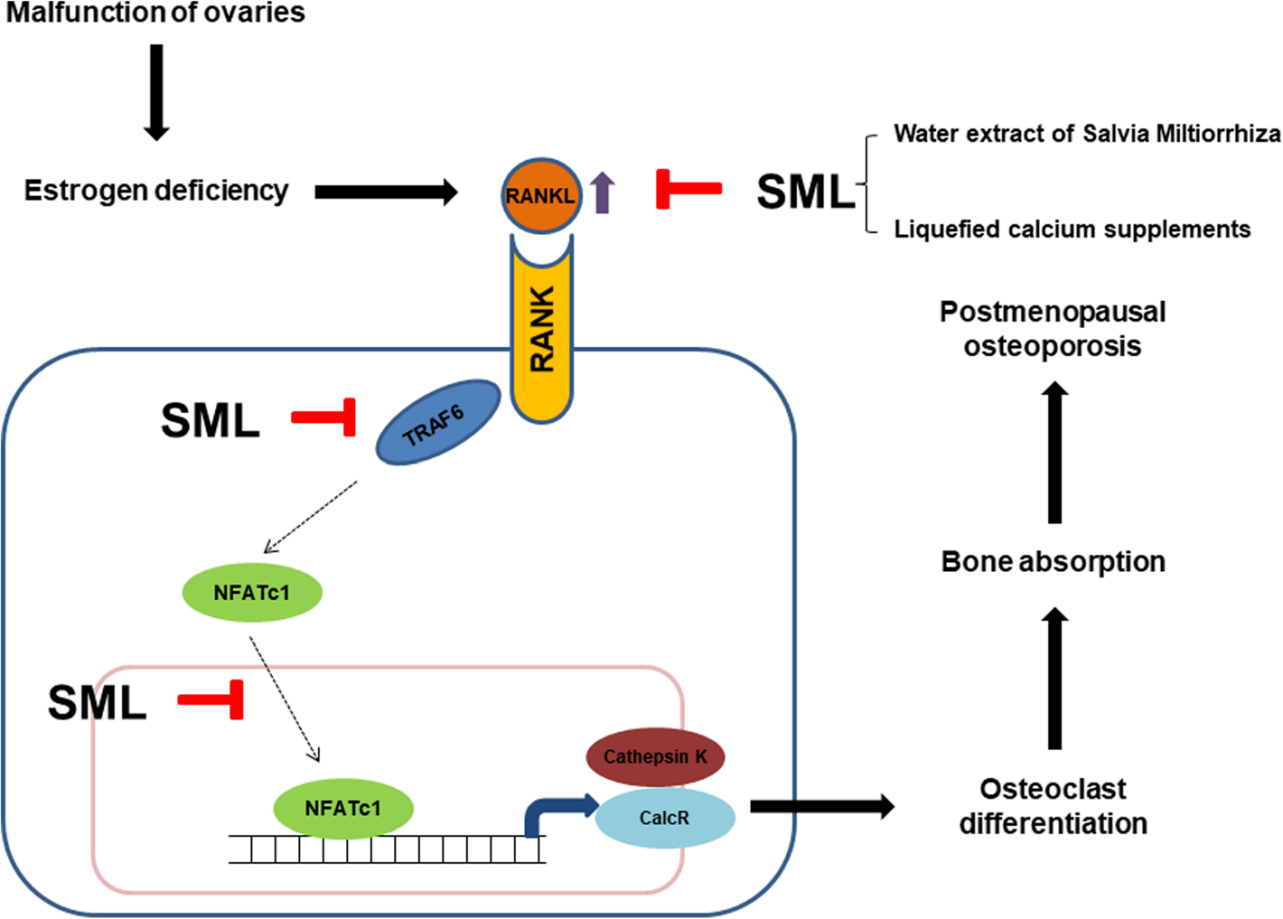
Schematic diagram showing the inhibitory effects of SML on OVX-induced osteoporosis via regulation of RANK/RANKL/OPG signaling pathways

In conclusion, these data suggest that SML has the protective effect on estrogen-deficient bone loss through blocking the RANKL signaling pathway-induced expression of TRAF6 and NFTAc1 as well as the upregulated cathepsin K and calcitonin receptor to promote osteoclast differentiation (Fig. 7). The inhibitory activity of SML in OVX mice implies the possibility of a pharmacological intervention specifically directed toward a disease like osteoporosis, where decreased bone strength increases the risk of a broken bone.

## Additional file

Additional file 1: Figure S1. HPLC analysis of SME and standards of Salvianolic acid. (**a**) HPLC chromatogram of water extract of *Salvia miltorrhiza* Bunge at 280 nm (**b**) HPLC results of standard salvianolic acid from the *Salvia miltorrhiza* Bunge (DOCX 25 kb)

## Abbreviations

BALP: Bone alkaline phosphatase
BMD: Bone volume fraction
BS/BV: Specific bone surface
BV: Bone volume fraction
BV/TV: Bone volume fraction
CalcR: Calcitonin receptor
cathepsin K: calcitonin receptor
Cs.Th: cross section thickness
E2: 17β-estradiol
HRT: Hormone replacement therapy
LCa: Liquefied calcium
MITF: Microphthalmia-associated transcription factor
MMI: Mean polar moment of inertia
NFTAc1: Nuclear factor of activated T-cells, cytoplasmic 1
OPG: Osteoprotegerin
OSCAR: Osteoclast-associated receptor
OVX: Ovariectomized
RANKL: Receptor activator of nuclear factor kappa-B ligand
ROS: Reactive oxygen species
SME: Ethanol extract of *S. miltiorrhiza* Bunge
SMI: Structure model index
SML: Combination of SME and LCa
Tb.N: Trabecular number
Tb.Pf: Trabecular bone pattern factor
Tb.Sp: Trabecular separation
Tb.Th: Trabecular thickness
TLC: Thin layer chromatography
TRAF6: TNF receptor associated factor 6
TRAP: Tartrate-resistant acid phosphatase
