# Supplementary material for: Effects of Salvia miltiorrhiza extract with supplemental liquefied calcium on osteoporosis in calcium-deficient ovariectomized mice
Source: BMC Complement Altern Med. 2017 Dec 20;17:545. doi: 10.1186/s12906-017-2047-y (PMC5738837; doi:10.1186/s12906-017-2047-y)
Supplement: Additional file 1: Figure S1. — HPLC analysis of SME and standards of Salvianolic acid. (a) HPLC chromatogram of water extract of Salvia miltorrhiza Bunge at 280 nm (b) HPLC results of standard salvianolic acid from the Salvia miltorrhiza Bunge (DOCX 25 kb) [file 12906_2017_2047_MOESM1_ESM.docx]

**File name:** Supplemental Information

**Title of data:** HPLC analysis of *Salvia miltiorrhiza* extract (SME) and salvianolic acid

**Description of data:** The SME was analyzed by HPLC chromatogram. Six principal SME peaks were detected in the chromatogram by diode array detection (DAD) at 280 nm. The highest peak was identified as salvianolic acid by comparison with the HPLC retention times of our standard compounds.


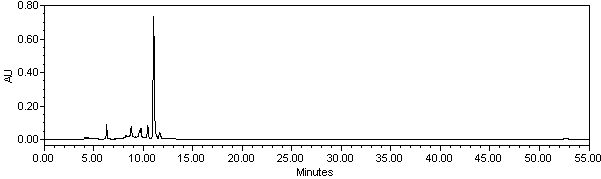

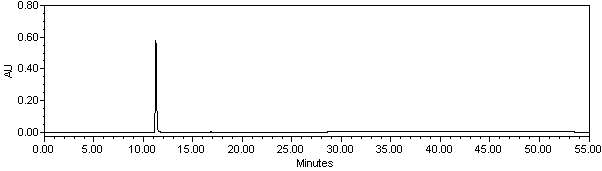


**a**

**b**

**Figure S1. HPLC analysis of SME and standards of Salvianolic acid. (a)** HPLC chromatogram of water extract of *Salvia miltorrhiza* Bunge at 280nm **(b)** HPLC results of standard salvianolic acid from the *Salvia miltorrhiza* Bunge.

**Supplemental Experimental procedures**

**HPLC analysis of SME and salvianolic acid**

An SIL-20A equipped with an SPD-M20A PDA and CTO-20A column oven (Shimadzu Corp., Kyoto, Japan) was used for HPLC analysis. Data were analyzed using the attached software. The fractions were separated at 40℃ on a YMC-pack J’sphere ODS-H80 (YMC, Tokyo, Japan) using a one-step linear gradient with mobile phase A [0.1% formic acid in water] and B [0.1% formic acid in acetonitrile:methanol (75:25, v/v)] . The gradient program began with 25% B and was held at this concentration for the first minute. This was followed by 40% B for 14 minutes, 60% A for 22 minutes, and 89% B for 25 minutes. It was then reduced to 25% B for 25 minutes. Salvianolic acid content was analyzed under the established HPLC conditions. Of the constituents in SME, salvianolic acid was found at the highest level (6.81%).
